# Parent–Child Concordance in Mediterranean Diet Adherence and Fast-Food Consumption: An Exploratory Study of Portuguese Families

**DOI:** 10.3390/nu18183050

**Published:** 2026-09-18

**Authors:** Leandro Oliveira, Marta Esgalhado

**Affiliations:** CBIOS (Research Center for Biosciences and Health Technologies), ECTS (School of Health Sciences and Technologies), Lusófona University, Campo Grande 376, 1749-024 Lisboa, Portugal

**Keywords:** Mediterranean Diet, MEDAS, KIDMED, fast food, parent–child concordance, dietary behaviour, children, family, Portugal

## Abstract

**Background/Objectives:** Parent–child dietary resemblance is well documented, but concordance in Mediterranean Diet adherence and fast-food consumption within the same family dyads remains underexplored in Portugal. This exploratory study examined parent–child associations across these complementary dietary dimensions. **Methods:** This cross-sectional study included 85 parent/guardian–child dyads recruited by convenience from two Portuguese basic education schools. Parents/guardians completed an online questionnaire for themselves and their child. Mediterranean Diet adherence was assessed using MEDAS and KIDMED. Associations were examined using Spearman correlations with bootstrap 95% confidence intervals, and fast-food concordance using exact agreement and weighted Cohen’s kappa. Multivariable linear and ordinal logistic regression models were also fitted. **Results:** Parental MEDAS was positively associated with child KIDMED (rho = 0.283, 95% CI 0.080–0.482, *p* = 0.009) and inversely with child fast-food consumption (rho = −0.304, 95% CI −0.491 to −0.094, *p* = 0.005). Parental fast-food consumption was inversely associated with child KIDMED (rho = −0.480, 95% CI −0.643 to −0.284, *p* < 0.001) and positively with child fast-food consumption (rho = 0.724, 95% CI 0.571–0.845, *p* < 0.001). Exact fast-food agreement was 76.5%, with quadratic-weighted kappa = 0.579 (95% CI 0.373–0.759). Adjusted analyses supported associations of parental MEDAS and child age with KIDMED, and parental fast-food frequency with child fast-food consumption. **Conclusions:** Parent–child dietary behaviours showed concordance across both dimensions, although Mediterranean Diet score concordance was modest. Larger longitudinal studies with direct assessment of both generations are needed to clarify direction and mechanisms in Portugal.

## 1. Introduction

Dietary behaviours established during childhood and adolescence are relevant to nutritional quality and may track, albeit imperfectly, into later stages of life [1,2]. Parents and caregivers share food environments, meals, purchasing decisions, and household practices with children, creating opportunities for dietary resemblance. Nevertheless, systematic evidence indicates that parent–child dietary associations are generally weak to moderate and vary according to the dietary component, population, developmental stage, and assessment method [1,2].

The Mediterranean Diet is particularly relevant in Southern European countries such as Portugal and is characterised by high consumption of plant foods and olive oil, moderate consumption of fish and other animal-source foods, and limited consumption of foods rich in saturated fat and added sugars [3,4]. Greater adherence has been associated with favourable health indicators across the lifespan [5,6,7,8]. However, Mediterranean countries are not uniformly characterised by high adherence, and traditional dietary practices coexist with contemporary food environments in which convenient, energy-dense, and highly processed foods are widely available [9,10].

Fast-food consumption provides a complementary behavioural marker because it is associated with higher energy, fat, sugar, and sugar-sweetened beverage intake and lower fruit and vegetable consumption among children and adolescents [11,12]. Family meal practices, home food availability, cooking frequency, parental dietary behaviour, and food purchasing may be associated with both overall diet quality and fast-food consumption [13,14,15,16]. Recent dyadic studies have also reported parent–child similarities in ultra-processed food intake and specific food categories [17,18,19].

Developmental stage may modify these relationships. As children progress through adolescence, greater autonomy over what, where, and with whom they eat increases exposure to peers, school settings, commercial food environments, and independent purchasing opportunities [20,21]. Thus, parent–child concordance may reflect a combination of shared household context, correlated behaviours, and broader environmental exposures rather than a simple unidirectional parental effect.

Association and agreement are also conceptually distinct: correlation describes the strength of a relationship, whereas agreement concerns the extent to which measurements correspond [22]. Parent–child dietary research has predominantly focused on associations, and systematic reviews have highlighted substantial methodological heterogeneity [1,2]. Examining both association and agreement may therefore provide a more complete description of dyadic similarity in an ordinal behaviour such as fast-food consumption.

Despite extensive evidence on familial dietary resemblance, Mediterranean Diet adherence and fast-food-related behaviours have often been examined separately. Their joint assessment within the same parent/guardian–child dyads remains comparatively underexplored in Portuguese families, a context in which the Mediterranean Diet remains an important cultural and nutritional reference while less traditional food behaviours are also present [23,24,25,26,27,28,29,30,31]. The present exploratory study therefore aimed to characterise Mediterranean Diet adherence and fast-food consumption in Portuguese parent/guardian–child dyads; examine associations across these dietary dimensions; quantify parent–child concordance in fast-food consumption; and explore demographic and socioeconomic factors associated with children’s dietary behaviours. We hypothesised that greater parental Mediterranean Diet adherence would be associated with greater adherence and lower fast-food consumption among children, and that higher parental fast-food consumption would be associated with more frequent child fast-food consumption.

## 2. Materials and Methods

### 2.1. Study Design and Participants

This exploratory observational cross-sectional study was conducted between February 2025 and June 2026 among parent/guardian–child dyads recruited from two basic education schools in Portugal. The schools were selected by convenience on the basis of feasibility and accessibility for recruitment. Families were invited through a QR code distributed to students to take home, which provided access to the online study questionnaire. Each participating parent/guardian–child dyad contributed data at a single time point, and the same questionnaire and data-collection procedure were used throughout the study period.

A total of 85 valid parent/guardian–child dyads were included in the analysis, and no completed responses were excluded. Eligibility for the parent/guardian comprised being aged 18 years or older, being the parent or legal guardian of a child attending basic education in Portugal, and agreeing to participate. The child was required to attend basic education in Portugal. The observed child age range of 8–17 years was not an a priori eligibility criterion. The total number of families receiving the invitation was not recorded; therefore, a participation rate could not be calculated.

No formal a priori sample size calculation was performed because the study was exploratory and recruitment was based on the number of eligible dyads available during the study period. Post hoc sensitivity calculations were used only to characterise the effects detectable with the achieved sample. With 85 dyads, a two-sided alpha of 0.05 and 80% power corresponded to a detectable correlation of approximately |r| = 0.30. For the multiple linear regression model with five predictors, the achieved sample corresponded to 80% power for an overall effect size of approximately Cohen’s f2 = 0.16. These calculations were not used to retrospectively redefine the study as confirmatory.

The study was reported in accordance with the Strengthening the Reporting of Observational Studies in Epidemiology (STROBE) guidelines for cross-sectional studies [32].

### 2.2. Data Collection

Data were collected through an online structured questionnaire accessed via the QR code provided to families. The participating parent or legal guardian completed all study instruments, reporting their own sociodemographic characteristics, anthropometric data, Mediterranean Diet adherence, and fast-food consumption, as well as information concerning the child, including age, sex, dietary behaviours, Mediterranean Diet adherence, and fast-food consumption. Thus, all child-related information was proxy-reported by the parent or legal guardian. Each dyad was assessed once. Participation was voluntary, questionnaires were anonymous, and the same assessment procedure was used throughout the recruitment period.

### 2.3. Sociodemographic and Anthropometric Characteristics

Sociodemographic information collected for parents/guardians included sex, age, marital status, educational level, approximate monthly net household income in euros, and household size. Educational attainment was originally collected using detailed educational levels and grouped for analysis as basic education (1st, 2nd, or 3rd cycle), secondary/professional education (secondary education or professional course), and higher education (bachelor’s, master’s, or doctoral degree). Marital status was retained as married, single, divorced, or cohabiting. Household size and household income were analysed as continuous variables. Income was available for 68 participants (80.0%). Parents/guardians self-reported their body weight and height, from which BMI was calculated and classified according to World Health Organization adult criteria [33,34]. Child weight and height were also requested by proxy report; however, these data were not analysed because responses included inconsistent units and implausible entries, and age was available only in completed years rather than the more precise age information required for appropriate age- and sex-specific paediatric BMI classification. For children and adolescents, age and sex were therefore used in the present analyses.

### 2.4. Mediterranean Diet Adherence

#### 2.4.1. Parents/Guardians

Mediterranean Diet adherence among parents/guardians was assessed using the 14-item Mediterranean Diet Adherence Screener (MEDAS), originally developed within the PREDIMED study [35,36]. The MEDAS evaluates key components of the Mediterranean dietary pattern, including olive oil use and consumption, vegetables, fruit, red and processed meat, butter, margarine or cream, sugar-sweetened beverages, wine, legumes, fish and seafood, commercial sweets and pastries, nuts, preference for poultry over red or processed meat, and sofrito. Each criterion met was assigned one point, resulting in a total score ranging from 0 to 14, with higher scores indicating greater adherence to the Mediterranean Diet. For descriptive purposes, MEDAS adherence was classified as low (≤5 points), moderate (6–9 points), or high (≥10 points). Individual MEDAS components were also examined descriptively.

#### 2.4.2. Children and Adolescents

Mediterranean Diet adherence among children and adolescents was assessed using the validated Portuguese version of the Mediterranean Diet Quality Index for Children and Adolescents (KIDMED) [37]. The instrument was administered without modification and completed by the participating parent or legal guardian with reference to the child’s dietary behaviours. The KIDMED comprises 16 dichotomous items; favourable behaviours contribute +1 point, and unfavourable behaviours contribute −1 point, yielding a total score from −4 to 12. Adherence was classified using the original criteria as low (≤3 points), intermediate (4–7 points), or high (≥8 points). Individual components were also examined descriptively. Because KIDMED includes a negatively scored item concerning weekly fast-food restaurant use, a sensitivity analysis recalculated the score after removing this item when examining associations between fast-food consumption frequency and KIDMED.

### 2.5. Fast-Food Consumption

Fast-food consumption was assessed using two study-specific questions that were not part of a validated instrument. Parents/guardians were asked, ‘How often do you eat meals at fast-food restaurants?’ and, separately, ‘How often does your child eat meals at fast-food restaurants?’ The parental response options were never, once per month or less, 2–3 times per month, 1–2 times per week, and 5–6 times per week; child response options were never, once per month or less, 2–3 times per month, and 1–2 times per week. No explicit recall period was specified, and no formal reliability or validity assessment of these study-specific items was conducted. For analyses requiring direct parent–child comparison, parental responses were harmonised into the same four ordered categories as the child variable: never, ≤1 time/month, 2–3 times/month, and ≥1 time/week. The original responses were otherwise retained. The KIDMED fast-food item was treated as a separate component of the validated KIDMED instrument and was not used to redefine responses to the study-specific fast-food question.

Information on family meals at fast-food restaurants was additionally collected for descriptive purposes.

### 2.6. Parent–Child Dietary Relationships

Parent–child dietary relationships were investigated across Mediterranean Diet adherence and fast-food consumption. Associations were examined between parental MEDAS scores and child KIDMED scores, parental MEDAS scores and child fast-food consumption frequency, parental fast-food consumption frequency and child KIDMED scores, and parental and child fast-food consumption frequencies.

For fast-food consumption, both association and agreement were examined because these represent distinct characteristics of dyadic data. Agreement was evaluated after harmonisation of parent and child frequency categories, as described above.

### 2.7. Statistical Analysis

Statistical analyses were performed using R version 4.6.1 (R Foundation for Statistical Computing, Vienna, Austria).

Continuous variables were summarised using mean and standard deviation (SD) when appropriate or median and interquartile range (IQR; 25th–75th percentile) for non-normally distributed variables. Categorical variables were presented as absolute and relative frequencies [*n* (%)].

Given the ordinal nature of several variables and the distributions of the dietary scores, non-parametric methods were used for bivariate analyses. Differences between two independent groups were assessed using the Wilcoxon rank-sum test, whereas comparisons across three or more groups were performed using the Kruskal–Wallis test. When a statistically significant Kruskal–Wallis test was observed, pairwise Wilcoxon rank-sum tests with Holm adjustment for multiple comparisons were performed.

Associations between continuous or ordinal variables were assessed using Spearman’s rank correlation coefficient (rho). To provide measures of precision, percentile bootstrap 95% confidence intervals for the principal correlation coefficients were estimated using 5000 resamples at the dyad level. Parent–child analyses examined parental MEDAS versus child KIDMED, parental MEDAS versus child fast-food frequency, parental fast-food frequency versus child KIDMED, and parental versus child fast-food frequency. Because KIDMED contains a fast-food item, correlations involving fast-food frequency and KIDMED were repeated after removing that item from the KIDMED total score as a sensitivity analysis.

Associations between parental and household characteristics and children’s dietary outcomes were additionally explored. Parental age, household income, and parental BMI were examined in relation to child KIDMED score and child fast-food consumption frequency using Spearman’s rank correlation. Differences in these outcomes according to parental educational level and marital status were assessed using the Kruskal–Wallis test. Differences in MEDAS scores and fast-food consumption according to parent/guardian sex, and in KIDMED scores and fast-food consumption according to child sex, were assessed using the Wilcoxon rank-sum test.

Parent–child agreement in harmonised fast-food consumption frequency was quantified using exact agreement and weighted Cohen’s kappa. Quadratic weights were used a priori because the categories were ordinal and disagreements spanning more categories were considered more substantial than adjacent-category disagreements. Bootstrap 95% confidence intervals were obtained from 5000 dyad-level resamples. Linear-weighted kappa was additionally calculated as a sensitivity analysis.

A multivariable linear regression model was fitted with child KIDMED total score as the dependent variable. Although KIDMED is discrete and bounded, its 17 possible values were treated as a quasi-continuous outcome for this exploratory model. Parental MEDAS score, parental educational level, child sex, and child age were included as predictors. Residual normality was assessed using the Shapiro–Wilk test, homoscedasticity using the Breusch–Pagan test, model specification using the Ramsey RESET test, and influential observations using Cook’s distance and DFBETAs. Multicollinearity was assessed using variance inflation factors (VIFs), with generalised VIFs adjusted for degrees of freedom for multi-level predictors. The results are reported as unstandardised coefficients (B), standard errors (SE), 95% confidence intervals, and *p*-values.

A multivariable ordinal logistic regression model with a cumulative logit link was fitted with child fast-food consumption frequency as the ordered dependent variable. Harmonised parental fast-food consumption frequency, child age, child sex, and parental educational level were included as predictors. The proportional-odds assumption was assessed using likelihood-ratio tests of nominal effects for each predictor, and multicollinearity was assessed using VIFs. As a sensitivity analysis, parental fast-food consumption was alternatively parameterised as a categorical factor and compared with the ordinal specification using a likelihood-ratio test. The results are reported as regression coefficients (B), odds ratios (OR), 95% confidence intervals, and *p*-values.

No imputation of missing data was performed. Bivariate analyses used available cases for each variable, whereas multivariable analyses used complete cases for the variables included in each model. Both primary multivariable models included all 85 dyads. Where sample size differed because of missing data, the corresponding *n* is reported.

All statistical tests were two-sided, and statistical significance was established at *p* < 0.05. Given the exploratory nature of the study, findings were interpreted as hypothesis-generating, with consideration given to the magnitude and direction of observed associations in addition to statistical significance.

### 2.8. Ethical Considerations

The study was conducted in accordance with the Declaration of Helsinki and its subsequent amendments and was approved by the Ethics Committee of the School of Health Sciences and Technologies of Lusófona University (reference no. P12–22, 8 February 2023). The participating parent or legal guardian provided informed consent before completing the questionnaire. Children and adolescents did not directly complete any study instrument in the dataset analysed here; all child-related information was proxy-reported by the parent or legal guardian. Separate child/adolescent assent was not collected, in accordance with the study protocol reviewed and approved by the Ethics Committee. Data were collected anonymously and used exclusively for research purposes.

## 3. Results

### 3.1. Participant Characteristics

A total of 85 parent/guardian–child dyads were analysed. Parents/guardians were predominantly female (67.1%), had a median age of 44 years (IQR 39–48; *n* = 78), and 54.1% had higher education. Children had a mean age of 12.2 ± 1.9 years (range 8–17), and 63.5% were male. Additional sociodemographic and parental anthropometric characteristics are presented in Table 1.

### 3.2. Mediterranean Diet Adherence and Fast-Food Consumption

Parents/guardians had a median MEDAS score of 7 (IQR 5–8), with moderate adherence most frequent (58.8%). Their fast-food consumption was generally infrequent, although 70.6% reported family meals at fast-food restaurants. Full distributions are presented in Table 2.

Children had a median KIDMED score of 8 (IQR 6–10); 58.8% had high, 30.6% intermediate, and 10.6% low adherence. Child fast-food consumption was most commonly ≤1 time/month (47.1%) or 2–3 times/month (37.6%) (Table 2).

### 3.3. Parent–Child Associations and Concordance in Dietary Behaviours

Individual MEDAS and KIDMED components are presented in Supplementary Table S1. The principal parent–child associations are summarised in Table 3. Parental MEDAS was positively associated with child KIDMED (rho = 0.283, 95% CI 0.080–0.482, *p* = 0.009) and inversely associated with child fast-food consumption (rho = −0.304, 95% CI −0.491 to −0.094, *p* = 0.005). Harmonised parental fast-food consumption was inversely associated with child KIDMED (rho = −0.480, 95% CI −0.643 to −0.284, *p* < 0.001) and positively associated with child fast-food consumption (rho = 0.724, 95% CI 0.571–0.845, *p* < 0.001).

After harmonisation into four ordered categories, exact parent–child agreement in fast-food consumption was 76.5%. Quadratic-weighted Cohen’s kappa was 0.579 (95% CI 0.373–0.759, *p* < 0.001); the linear-weighted sensitivity estimate was similar (kappa = 0.603, 95% CI 0.432–0.753, *p* < 0.001). In sensitivity analyses excluding the fast-food item from KIDMED, inverse associations remained for parental fast-food consumption (rho = −0.422, 95% CI −0.594 to −0.221, *p* < 0.001) and child fast-food consumption (rho = −0.363, 95% CI −0.551 to −0.148, *p* < 0.001).

### 3.4. Parental and Household Factors Associated with Children’s Dietary Behaviours

Exploratory associations between parental/household characteristics and child dietary outcomes are also presented in Table 3. Household income was positively associated with child KIDMED (rho = 0.381, *p* = 0.001) and inversely associated with child fast-food consumption (rho = −0.249, *p* = 0.040). Parental BMI was positively associated with child fast-food consumption (rho = 0.226, *p* = 0.038).

Child KIDMED differed overall by parental educational level (H = 6.38, *p* = 0.041), although Holm-adjusted pairwise comparisons were not significant. Child fast-food consumption did not differ significantly by parental education (H = 5.16, *p* = 0.076), and parental age and marital status were not associated with either child outcome (Table 3).

### 3.5. Multivariable Factors Associated with Children’s Dietary Behaviours

Multivariable analyses were conducted to identify factors independently associated with children’s Mediterranean Diet adherence and fast-food consumption frequency.

In the linear model, parental MEDAS was positively associated with child KIDMED (B = 0.371, 95% CI 0.076–0.666, *p* = 0.014), whereas child age was inversely associated (B = −0.326, 95% CI −0.639 to −0.013, *p* = 0.041). The model explained 21.7% of the variance (adjusted R^2^ = 0.168) (Table 4). Diagnostics did not indicate material violations: Shapiro–Wilk W = 0.976 (*p* = 0.115), Breusch–Pagan (*p* = 0.150), RESET (*p* = 0.056). Furthermore, adjusted GVIF values ranged from 1.008 to 1.066. No observation had Cook’s distance approaching 1; excluding the five observations above the conventional 4/n screening threshold yielded similar estimates for parental MEDAS and child age.

In the ordinal logistic model, each one-category increase in harmonised parental fast-food consumption was associated with higher odds of the child being in a higher fast-food consumption category (OR = 21.98, 95% CI 8.80–54.90, *p* < 0.001) (Table 5). Child age, sex, and parental education were not independently associated with the outcome. There was no statistically significant evidence of violation of the proportional-odds assumption for parental fast-food consumption (*p* = 0.235), child age (*p* = 0.537), child sex (*p* = 0.083), or parental education (*p* = 0.642). VIF values ranged from 1.04 to 1.07. Treating parental fast-food consumption as a categorical factor did not improve model fit compared with the ordinal specification (likelihood-ratio chi-square (2) = 0.13, *p* = 0.935).

## 4. Discussion

This exploratory study indicates that Mediterranean Diet adherence and fast-food consumption cluster within Portuguese parent/guardian–child dyads, but with different magnitudes. Concordance was modest for Mediterranean Diet adherence and considerably greater for fast-food consumption. These patterns should be interpreted as associations within a shared family and social context rather than evidence of a unidirectional parental effect.

The generally favourable KIDMED distribution is broadly consistent with previous Portuguese studies, although adherence estimates vary by age and setting [23,24,25]. The inverse association between age and KIDMED also aligns with evidence that diet quality and adherence to traditional Mediterranean practices may decline as children move into adolescence [38,39,40,41,42]. Greater dietary autonomy, peer influence, school and commercial food environments, and independent purchasing may contribute to this developmental pattern [43,44]. Longitudinal evidence suggests that diet quality and broader dietary patterns show moderate tracking from childhood into adolescence and adulthood, while also allowing substantial individual change over time [45,46]. However, current evidence does not establish that adolescents who move away from a Mediterranean pattern will predictably return to it later in life. This remains an important question for longitudinal research.

Among parents/guardians, the predominance of moderate rather than high Mediterranean Diet adherence is compatible with evidence that adherence to the traditional pattern is not uniformly high in Portugal or other Mediterranean settings [26,27,28,29,30,31]. The coexistence of traditional components, such as olive oil use, with less favourable behaviours illustrates why Mediterranean Diet adherence and fast-food consumption can be informative as complementary rather than mutually exclusive dimensions of family diet.

The positive parent–child association in Mediterranean Diet adherence was modest, which is consistent with meta-analytic evidence showing generally moderate familial resemblance for whole-diet measures [2]. Similar associations have been reported in other Mediterranean populations [38,47,48]. Potential explanations include shared food availability, meal routines, purchasing and preparation practices, and behavioural modelling [49], but these mechanisms were not measured directly here and should therefore be regarded as hypotheses rather than demonstrated pathways.

Fast-food consumption showed a clearer dyadic pattern. Its association with child KIDMED persisted even when the fast-food item was removed from the KIDMED score, indicating that the finding was not solely a mathematical consequence of item overlap. Nevertheless, the attenuation observed in this sensitivity analysis confirms that the overlap should be considered when interpreting associations between fast-food frequency and the complete KIDMED score. Convenience, affordability, time constraints, food availability, and habitual purchasing may plausibly contribute to shared fast-food behaviours [49,50,51,52], although the present design cannot identify which mechanisms are responsible.

The relatively high correlation and moderate-to-substantial weighted agreement for fast-food consumption provide complementary evidence that parent and child frequencies tend to co-vary within households. The adjusted ordinal model was consistent with this pattern, but its large odds ratio and wide confidence interval indicate considerable uncertainty in the magnitude of the association. Previous studies have also reported relationships between parental and child or adolescent fast-food consumption [52,53,54]. Because all child information was proxy-reported by the same parent/guardian who reported their own behaviour, shared-method effects may have increased apparent concordance and should temper interpretation.

The direction of these relationships cannot be determined from cross-sectional data. During adolescence, children may increasingly contribute to restaurant choices and household food requests while simultaneously being exposed to peers, school environments, marketing, digital media, and independent purchasing opportunities [43,44,55]. Health-related dietary requirements of a child, including food allergies, intolerances, or other medically indicated restrictions, may also prompt changes in household purchasing and meal preparation. These possibilities illustrate the potentially reciprocal nature of family dietary behaviour, but they were not assessed in the present study.

The exploratory socioeconomic findings are broadly compatible with literature linking family socioeconomic circumstances to children’s diet quality, although results vary across populations and indicators [39,42,56]. Food affordability, nutrition knowledge, working patterns, time available for meal preparation, and access to food outlets may all contribute. Household income was missing for 20% of participants and was not included in the primary multivariable models, so these associations should not be interpreted as independent socioeconomic effects.

No clear sex-related differences emerged, consistent with heterogeneous evidence regarding sex and Mediterranean Diet adherence [23,39,41,57,58]. In contrast, the age association reinforces the importance of developmental context. The persistence of the parental MEDAS association after adjustment for age suggests that parent–child dietary resemblance can coexist with increasing adolescent autonomy, rather than demonstrating that parental behaviour determines the child’s diet.

From a public health perspective, the findings identify the family context as a relevant setting for further investigation rather than demonstrating the effectiveness of any specific family-based intervention. Future intervention development may benefit from considering household food availability, purchasing, meal routines, and eating outside the home alongside influences that become increasingly important in adolescence, such as peers, schools, marketing, digital media, and the wider food environment [43,55]. Prospective and intervention studies are needed before specific policy or programme recommendations can be made.

### Strengths, Limitations, and Future Research

Strengths include the dyadic design, use of age-appropriate Mediterranean Diet instruments, complementary assessment of association and agreement for fast-food consumption, bootstrap confidence intervals for the principal dyadic estimates, and sensitivity analyses addressing both weighting of kappa and overlap between the KIDMED and fast-food measures.

Several limitations should be considered. The cross-sectional design precludes conclusions about temporality or causality. The convenience sample of 85 dyads from two schools limits generalisability and precision, particularly for subgroup and multivariable analyses; this is reflected in the wide confidence interval around the parental fast-food estimate. The number of families receiving the invitation was not recorded, so a participation rate could not be calculated and selection bias cannot be excluded.

All dietary and sociodemographic information was reported by one parent or legal guardian. Child dietary behaviours were therefore proxy-reported, and the same respondent reported both their own and the child’s fast-food consumption, creating potential recall, social desirability, and shared-method biases. Moreover, one participating parent or guardian may not represent the dietary behaviour of the entire household. Partners or other caregivers may have different diets, eating schedules, food-related responsibilities, and patterns of eating outside the home, particularly because of work or other obligations.

The study-specific fast-food questions had no predefined recall period and had not undergone formal reliability or validity testing. In addition, the KIDMED contains a fast-food item, creating partial conceptual and mathematical overlap with the separate fast-food variable. The sensitivity analysis excluding this item reduced but did not eliminate the observed associations. MEDAS and KIDMED are also distinct instruments with different items and scoring systems, so their association reflects resemblance in the broader construct of Mediterranean Diet adherence rather than agreement between equivalent measures.

Although child weight and height were requested, proxy-reported anthropometric data contained inconsistent units and implausible entries and could not be used reliably. In addition, age was recorded only in completed years, limiting appropriate age- and sex-specific paediatric BMI classification. Consequently, child nutritional status could not be examined in relation to the dietary outcomes.

Household income was missing for 17 participants (20.0%), and no imputation was performed. Other potentially relevant determinants, including parental feeding practices, home food availability, family meal characteristics, health-related dietary restrictions, peer influences, food purchasing autonomy, school food environments, and marketing exposure, were not comprehensively assessed. Residual confounding therefore remains possible.

Future studies should use larger, socioeconomically and geographically diverse samples and longitudinal designs to clarify the direction and evolution of parent–child dietary relationships. Independent assessment of both parents/caregivers and children, together with direct or observational measures of food purchasing, meal contexts, and dietary behaviour, would help assess concordance between reported and observed behaviours. Economic approaches examining household food expenditure, food prices, affordability, and purchasing patterns could further clarify socioeconomic mechanisms. Collecting information from multiple caregivers and using standardised measured anthropometry would provide a more complete representation of household dietary dynamics and nutritional status.

## 5. Conclusions

This exploratory study found parent–child concordance in Mediterranean Diet adherence and fast-food consumption among Portuguese families, with a modest association for Mediterranean Diet scores and a larger association for fast-food frequency. The findings suggest that the family context may be relevant to understanding children’s dietary behaviours, while also highlighting the importance of developmental, socioeconomic, and wider environmental influences. Because the study was cross-sectional, based on a small convenience sample, and relied on parent/guardian proxy reporting, causal or intervention conclusions are not warranted. Larger longitudinal studies with direct assessment of both generations are needed to clarify the direction, mechanisms, and practical implications of these associations.

## Figures and Tables

**Table 1:** Sociodemographic and anthropometric characteristics of parents/guardians and children.

Characteristic	Total (*n* = 85)
**Parents/Guardians**	
**Sex**, *n* (%)	
Female	57 (67.1)
Male	28 (32.9)
**Age** (years), median (IQR)	44 (39–48) ^1^
**Marital status**, *n* (%)	
Married	47 (55.3)
Single	26 (30.6)
Divorced	9 (10.6)
Cohabiting	3 (3.5)
**Educational level**, *n* (%)	
Basic education	17 (20.0)
Secondary/professional education	22 (25.9)
Higher education	46 (54.1)
**Household size**, median (IQR)	4 (3–4)
**Monthly household income** (€), median (IQR)	2200 (1500–3500) ^2^
**BMI** (kg/m^2^), median (IQR)	24.4 (22.3–27.1)
**BMI category**, *n* (%)	
Underweight	1 (1.2)
Normal weight	45 (52.9)
Overweight	31 (36.5)
Obesity	8 (9.4)
**Children**	
**Sex**, *n* (%)	
Female	31 (36.5)
Male	54 (63.5)
**Age** (years), mean ± SD	12.2 ± 1.9
Age range, years	8–17

Data are presented as absolute values (percentages) for categorical variables and as mean ± SD or median IQR for continuous variables, as appropriate. ^1^ Age data available for 78 parents/guardians. ^2^ Household income data available for 68 participants after data cleaning; implausible or uninterpretable responses were treated as missing. BMI, body mass index; IQR, interquartile range; SD, standard deviation.

**Table 2:** Mediterranean Diet adherence and fast-food consumption among parents/guardians and children.

Variable	Data
Parents/guardians	
MEDAS score, median (IQR)	7 (5–8)
MEDAS adherence category, *n* (%)	
Low adherence	24 (28.2)
Moderate adherence	50 (58.8)
High adherence	11 (12.9)
Fast-food consumption frequency, *n* (%)	
Never	8 (9.4)
≤1 time/month	40 (47.1)
2–3 times/month	31 (36.5)
≥1 time/week	6 (7.1)
Family meals at fast-food restaurants, *n* (%)	
No	25 (29.4)
Yes	60 (70.6)
Children	
KIDMED score, median (IQR)	8 (6–10)
KIDMED adherence category, *n* (%)	
Low adherence	9 (10.6)
Intermediate adherence	26 (30.6)
High adherence	50 (58.8)
Fast-food consumption frequency, *n* (%)	
Never	7 (8.2)
≤1 time/month	40 (47.1)
2–3 times/month	32 (37.6)
1–2 times/week	6 (7.1)

Data are presented as *n* (%) or median (IQR), as appropriate. MEDAS adherence was classified as low (≤5 points), moderate (6–9 points), or high (≥10 points). KIDMED adherence was classified as low (≤3 points), intermediate (4–7 points), or high (≥8 points). IQR, interquartile range; KIDMED, Mediterranean Diet Quality Index for Children and Adolescents; MEDAS, Mediterranean Diet Adherence Screener.

**Table 3:** Parent–child dietary associations, agreement, and exploratory parental/household correlates.

Analysis	Outcome/Relationship	Measure	Estimate (95% CI)	*p*-Value
Parent–child	Parental MEDAS vs. child KIDMED	Spearman rho	0.283 (0.080 to 0.482)	0.009
Parent–child	Parental MEDAS vs. child fast-food	Spearman rho	−0.304 (−0.491 to −0.094)	0.005
Parent–child	Parental fast-food vs. child KIDMED	Spearman rho	−0.480 (−0.643 to −0.284)	<0.001
Parent–child	Parental vs. child fast-food	Spearman rho	0.724 (0.571 to 0.845)	<0.001
Agreement	Parent–child fast-food	Exact agreement	76.5%	
Agreement	Parent–child fast-food	Quadratic-weighted kappa	0.579 (0.373 to 0.759)	<0.001
Sensitivity	Parental fast-food vs. KIDMED without FF item	Spearman rho	−0.422 (−0.594 to −0.221)	<0.001
Sensitivity	Child fast-food vs. KIDMED without FF item	Spearman rho	−0.363 (−0.551 to −0.148)	< 0.001
Parental/household	Household income vs. child KIDMED	Spearman rho	0.381	0.001
Parental/household	Household income vs. child fast-food	Spearman rho	−0.249	0.040
Parental/household	Parental BMI vs. child KIDMED	Spearman rho	−0.073	0.509
Parental/household	Parental BMI vs. child fast-food	Spearman rho	0.226	0.038
Parental/household	Parental education vs. child KIDMED	Kruskal–Wallis H	6.38	0.041
Parental/household	Parental education vs. child fast-food	Kruskal–Wallis H	5.16	0.076
Parental/household	Parental age vs. child KIDMED	Spearman rho	−0.053	0.629
Parental/household	Parental age vs. child fast-food	Spearman rho	−0.068	0.536
Parental/household	Marital status vs. child KIDMED	Kruskal–Wallis H	2.00	0.573
Parental/household	Marital status vs. child fast-food	Kruskal–Wallis H	0.95	0.814

BMI, body mass index; CI, confidence interval; FF, fast-food; H, Kruskal–Wallis statistic; KIDMED, Mediterranean Diet Quality Index for Children and Adolescents; MEDAS, Mediterranean Diet Adherence Screener. Bootstrap 95% CIs for principal Spearman correlations and weighted kappa used 5000 dyad-level resamples. Pairwise Wilcoxon tests after the significant education omnibus test were non-significant after Holm adjustment.

**Table 4:** Multivariable linear regression analysis of factors associated with children’s KIDMED scores.

Predictor	B	SE	95% CI	*p*-Value
Parental MEDAS score	0.371	0.148	0.076 to 0.666	0.014
Parental education: Secondary/professional ^1^	0.879	0.917	−0.947 to 2.705	0.341
Parental education: Higher education ^1^	1.581	0.837	−0.084 to 3.246	0.062
Child sex: Male ^2^	0.398	0.625	−0.847 to 1.642	0.527
Child age, years	−0.326	0.157	−0.639 to −0.013	0.041

B, unstandardised regression coefficient; SE, standard error; CI, confidence interval; MEDAS, Mediterranean Diet Adherence Screener; KIDMED, Mediterranean Diet Quality Index for Children and Adolescents. ^1^ Reference category: basic education. ^2^ Reference category: female. Model statistics: *n* = 85; R^2^ = 0.217; adjusted R^2^ = 0.168; F(5,79) = 4.386; *p* = 0.001.

**Table 5:** Multivariable ordinal logistic regression analysis of factors associated with children’s fast-food consumption frequency.

Predictor	B	SE	OR	95% CI	*p*-Value
Parental fast-food frequency	3.090	0.467	21.98	8.80–54.90	<0.001
Child age, years	0.164	0.133	1.18	0.91–1.53	0.215
Child sex: Male ^1^	−0.916	0.539	0.40	0.14–1.16	0.092
Parental education: Secondary/professional ^2^	−0.491	0.755	0.61	0.14–2.70	0.516
Parental education: Higher education ^2^	−0.514	0.674	0.60	0.16–2.25	0.447

B, regression coefficient; SE, standard error; OR, odds ratio; CI, confidence interval. ^1^ Reference category: female. ^2^ Reference category: basic education. Parental fast-food consumption was harmonised into four ordered categories. Proportional-odds tests: parental fast-food *p* = 0.235; child age *p* = 0.537; child sex *p* = 0.083; parental education *p* = 0.642.

## Data Availability

The data supporting the findings of this study are available from the corresponding author upon reasonable request. The data are not publicly available due to privacy and ethical considerations related to the participation of children and parent/guardian–child dyads.

## Supplementary Materials

The following supporting information can be downloaded at https://www.mdpi.com/article/10.3390/nu18183050/s1, Table S1. Individual components of Mediterranean Diet adherence among parents/guardians and children.

## Abbreviations

The following abbreviations are used in this manuscript:

BMI	Body Mass Index
CI	Confidence Interval
IQR	Interquartile Range
KIDMED	Mediterranean Diet Quality Index for Children and Adolescents
MEDAS	Mediterranean Diet Adherence Screener
OR	Odds Ratio
PREDIMED	Prevención con Dieta Mediterránea
SD	Standard Deviation
SE	Standard Error
STROBE	Strengthening the Reporting of Observational Studies in Epidemiology
WHO	World Health Organization
