# Supplementary material for: Parent–Child Concordance in Mediterranean Diet Adherence and Fast-Food Consumption: An Exploratory Study of Portuguese Families
Source: Nutrients. 2026 Sep 18;18(18):3050. doi: 10.3390/nu18183050 (PMC13610788; doi:10.3390/nu18183050)
Supplement: Supplementary file 1 [file nutrients-18-03050-s001.zip › nutrients-4567452-supplementary.pdf]

Supplementary Materials

Table S1. Individual components of Mediterranean Diet adherence among parents/guardians and children.

Item	Dietary component	n (%)
**MEDAS, parents/guardians**		
1	Olive oil as the main culinary fat	79 (92.9)
2	Olive oil consumption ≥4 tablespoons/day	25 (29.4)
3	Vegetables ≥2 servings/day	40 (47.1)
4	Fruit ≥3 servings/day	29 (34.1)
5	Red/processed meat <1 serving/day	36 (42.4)
6	Butter, margarine, or cream <1 serving/day	54 (63.5)
7	Sugar-sweetened/carbonated beverages <1 serving/day	49 (57.6)
8	Wine ≥7 glasses/week	17 (20.0)
9	Legumes ≥3 servings/week	36 (42.4)
10	Fish/seafood ≥3 servings/week	36 (42.4)
11	Commercial sweets or pastries <3 times/week	59 (69.4)
12	Nuts ≥3 servings/week	16 (18.8)
13	Preference for poultry over red/processed meat	67 (78.8)
14	Sofrito ≥2 times/week	46 (54.1)
**KIDMED, children**		
1	Consumes fruit or natural fruit juice daily	74 (87.1)
2	Consumes a second fruit daily	65 (76.5)
3	Consumes vegetables once daily	68 (80.0)
4	Consumes vegetables more than once daily	54 (63.5)
5	Consumes fish regularly	56 (65.9)
6	Goes to a fast-food restaurant ≥1 time/week	26 (30.6)
7	Consumes legumes >1 time/week	57 (67.1)
8	Consumes pasta or rice ≥5 days/week	75 (88.2)
9	Consumes cereals or cereal products at breakfast	70 (82.4)
10	Consumes nuts regularly	33 (38.8)
11	Uses olive oil at home	80 (94.1)
12	Eats breakfast daily	74 (87.1)
13	Consumes dairy products at breakfast	49 (57.6)

14	Consumes commercially baked goods/pastries at breakfast	23 (27.1)
15	Consumes two yogurts and/or 40 g of cheese daily	43 (50.6)
16	Consumes sweets/candy several times per day	17 (20.0)

Values are presented as absolute value (percentage, based on the total sample of 85 parent–child dyads. For MEDAS, frequencies indicate participants meeting the criterion corresponding to one point in the score. For KIDMED, frequencies indicate the reported dietary behaviours. Items 6, 14, and 16 represent unfavourable behaviours and are negatively scored, whereas item 12 is presented in its positive form (daily breakfast consumption), although breakfast skipping is negatively scored in the KIDMED index. MEDAS, Mediterranean Diet Adherence Screener; KIDMED, Mediterranean Diet Quality Index for children and adolescents.
